# Weathering the COVID-19 storm: The impact on health professionals

**DOI:** 10.4102/hsag.v26i0.1690

**Published:** 2021-10-21

**Authors:** Jennifer Chipps, Mary Ann Jarvis

**Affiliations:** 1 School of Nursing, Faculty of Community and Health Sciences, University of the Western Cape, Cape Town, South Africa; 2 Discipline of Nursing, School of Health Sciences, University of KwaZulu-Natal, Durban, South Africa

During the coronavirus disease 2019 (COVID-19) pandemic, health professionals have been on the forefront of delivering healthcare whilst experiencing unprecedented stress facing complex ethical situations, heavy workloads, long working hours and high levels of patient acuity and deaths (Fernandez-Parsons, Rodriguez & Goyal 2013; Talevi et al. 2020). Health professionals have continued to display a professional duty of care (Fernandez et al. 2020; Spoorthy, Pratapa & Mahant 2020; Valdez 2021) with, for example, reports of 97% of the frontline nurses in China expressing their willingness to work during the pandemic (Hu et al. 2020). This duty of care is embedded through professional education and socialisation of health professionals with expectations and norms about saving lives, relieving suffering and not abandoning patients (Turale, Meechamnan & Kunaviktikul 2020).

Duty of care has put health professionals at an increased risk of stress, with reports of poor mental health outcomes (Lai et al. 2020). Many health professionals became infected and died. In South Africa, by August 2020, the mortality rate because of COVID-19 amongst health professionals was 0.9% (Naidoo et al. 2020). More than half of the infections reported were amongst nurses (Naidoo et al. 2020) with nearly a quarter of South African nurse respondents in a national survey reporting that COVID-19 had impacted their health and well-being (Naidoo et al. 2020). These findings are consistent with global reports of high levels of psychological distress amongst health professionals (Hu et al. 2020; Lai et al. 2020; Shechter et al. 2020), with reports of moderate and high burnout with emotional exhaustion (60%), depersonalisation (42.3%), anxiety and depression (nearly 50%) (Hu et al. 2020).

Psychological distress is a response to stressors and during the pandemic stressors were heightened. A major source of stress was health professionals’ fears of safety for self and loved ones. In a national survey of health professionals in South Africa, nearly 80% reported that COVID-19 presented them with a greater risk of infection because of their profession and more than half of nurse practitioners expressed concerns about infecting loved ones (HSRC 2020). Even though dealing with the risks of infectious diseases is not new for health professionals, the novel nature of the COVID-19 pandemic and the changed working conditions with high and poorly understood risks presented major additional stresses (Hu et al. 2020).

Another source of stress during the pandemic was the rapid and at times, unpredictable changes occurring in the work environment. The COVID-19 work environment has been characterised by a cumulative mortality of patients and staff, exhaustion because of increased and changing workloads, anxiety over limited medical resources and personal protective equipment (PPE) and unexpected and challenging changes in staff skill mixes and role changes. During the pandemic, wards have been converted to care for COVID-19 patients, newly graduated staff suddenly found that they were the most senior staff on duty, and non-specialists have been rapidly trained in intensive care and allocated as primary care providers in specialist units (Catania et al. 2021). All these factors have exposed health professionals to medico-legal risks and psychological distress.

Duty of care and social and moral responsibilities of health professionals to continue to work during the outbreak have also contributed to psychological distress (Cai et al. 2020). The pandemic has presented health professionals with many distressing situations. They have been confronted with issues of fair allocation of resources and equity of care such as triage of equipment to those who have a better chance of survival, denying or delaying care, patients not expected to survive still needing care and patients dying without family because of visitor’s restrictions (Greenberg et al. 2020). In these situations, action or lack of action is often in conflict with professional values and can negatively impact feelings of professional integrity and identity (Morley et al. 2019). As a result, health professionals are at risk of experiencing moral distress and injury (Turale et al. 2020), increased psychological distress and undermining professional self-confidence (Reed & Rishel 2015).

However, despite all these challenges, there has been an emergence of health professional adaptation, resilience and growth. There have been reports of post-traumatic growth, with 61% of nurses in a study in China reporting a sense of personal accomplishment because of their work during the pandemic (Hu et al. 2020). Nurses in a study in Poland also reported positive changes resulting from painful experiences related to the pandemic (Nowicki et al. 2020), and nurses in Italy reportedly expressed a strong spirit of adaptation and resilience to the rapid changes and challenges they faced (Catania et al. 2021).

Health professionals require strong moral courage, stamina and resilience to weather the COVID-19 storm. Health service organisations should have work-related support initiatives in place through expanded and flexible employee assistance programmes to protect their staff both physically and emotionally (Catania et al. 2021; Dean, Jacobs & Manfredi 2020). Specific attention should also be given to appropriate professional role differentiation with adequate training and preparation of specialist staff. To ensure that health workers can weather future crises such as the COVID-19 pandemic, it is essential that these initiatives be embedded in the organisational culture.

## References

[CIT0001] Cai, H., Tu, B., Ma, J., Chen, L., Fu, L., Jiang, Y. et al., 2020, ‘Psychological impact and coping strategies of frontline medical staff in Hunan between January and March 2020 during the outbreak of coronavirus disease 2019 (COVID-19) in Hubei, China’, *Medical Science Monitor: International Medical Journal of Experimental and Clinical Research* 26, e924171. 10.12659/MSM.924171 32291383 PMC7177038

[CIT0002] Catania, G., Zanini, M., Hayter, M., Timmins, F., Dasso, N., Ottonello, G. et al., 2021, ‘Lessons from Italian front-line nurses’ experiences during the COVID-19 pandemic: A qualitative descriptive study’, *Journal of Nursing Management* 29(3), 404–411. 10.1111/jonm.13194 33107657

[CIT0003] Dean, W., Jacobs, B. & Manfredi, R.A., 2020, ‘Moral injury: The invisible epidemic in COVID health care workers’, *Annals of Emergency Medicine* 76(4), 385–386. 10.1016/j.annemergmed.2020.05.023 32586634 PMC7308739

[CIT0004] Fernandez-Parsons, R., Rodriguez, L. & Goyal, D., 2013, ‘Moral distress in emergency nurses’, *Journal of Emergency Nursing* 39(6), 547–552. 10.1016/j.jen.2012.12.009 23414814

[CIT0005] Fernandez, R., Lord, H., Halcomb, E., Moxham, L., Middleton, R., Alananzeh, I. et al., 2020, ‘Implications for COVID-19: A systematic review of nurses’ experiences of working in acute care hospital settings during a respiratory pandemic’, *International Journal of Nursing Studies* 111, 103637. 10.1016/j.ijnurstu.2020.103637 32919358 PMC7206441

[CIT0006] Greenberg, N., Docherty, M., Gnanapragasam, S. & Wessely, S., 2020, ‘Managing mental health challenges faced by healthcare workers during covid-19 pandemic’, *BMJ* 368, m1211. 10.1136/bmj.m1211 32217624

[CIT0007] HSRC, 2020, *Healthcare worker survey* [Online], HSRC, Durban, viewed 21 July 2021, from http://www.hsrc.ac.za/en/media-briefs/general/lockdown-survey-results

[CIT0008] Hu, D., Kong, Y., Li, W., Han, Q., Zhang, X., Zhu, L.X. et al., 2020, ‘Frontline nurses’ burnout, anxiety, depression, and fear statuses and their associated factors during the COVID-19 outbreak in Wuhan, China: A large-scale cross-sectional study’, *EClinicalMedicine* 24, 100424. 10.1016/j.eclinm.2020.100424 32766539 PMC7320259

[CIT0009] Lai, J., Ma, S., Wang, Y., Cai, Z., Hu, J., Wei, N. et al., 2020, ‘Factors associated with mental health outcomes among health care workers exposed to coronavirus disease 2019’, *JAMA Network Open* 3(3), e203976. 10.1001/jamanetworkopen.2020.3976 32202646 PMC7090843

[CIT0010] Morley, G., Ives, J., Bradbury-Jones, C. & Irvine, F., 2019, ‘What is ‘moral distress’? A narrative synthesis of the literature’, *Nursing Ethics* 26(3), 646–662 10.1177/0969733017724354 28990446 PMC6506903

[CIT0011] Naidoo, I., Mabaso, M., Moshabela, M., Sewpaul, R. & Reddy, S.P., 2020, ‘South African health professionals’ state of well-being during the emergence of COVID-19’, *South African Medical Journal* 110(10), 956. 10.7196/SAMJ.2020.v110i10.15250 33205717

[CIT0012] Nowicki, G.J., Ślusarska, B., Tucholska, K., Naylor, K., Chrzan-Rodak, A. & Niedorys, B., 2020, ‘The severity of traumatic stress associated with COVID-19 pandemic, perception of support, sense of security, and sense of meaning in life among nurses: Research protocol and preliminary results from Poland’, *International Journal of Environmental Research and Public Health* 17(18), 6491. 10.3390/ijerph17186491 PMC7559728 32906590

[CIT0013] Reed, P.G. & Rishel, C.J., 2015, ‘Epistemic injustice and nurse moral distress: Perspective for policy development’, *Nursing Science Quarterly* 28(3), 241–244. 10.1177/0894318415585634 26109704

[CIT0014] Shechter, A., Diaz, F., Moise, N., Anstey, D.E., Ye, S., Agarwal, S. et al., 2020, ‘Psychological distress, coping behaviors, and preferences for support among New York healthcare workers during the COVID-19 pandemic’, *General Hospital Psychiatry* 66, 1–8. 10.1016/j.genhosppsych.2020.06.007 32590254 PMC7297159

[CIT0015] Spoorthy, M.S., Pratapa, S.K. & Mahant, S., 2020, ‘Mental health problems faced by healthcare workers due to the COVID-19 pandemic – A review’, *Asian Journal of Psychiatry* 51, 102119. 10.1016/j.ajp.2020.102119 32339895 PMC7175897

[CIT0016] Talevi, D., Socci, V., Carai, M., Carnaghi, G., Faleri, S., Trebbi, E. et al., 2020, ‘Mental health outcomes of the COVID-19 pandemic’, *Rivista di Psichiatria* 55(3), 137–144. 10.1708/3382.33569 32489190

[CIT0017] Turale, S., Meechamnan, C. & Kunaviktikul, W., 2020, ‘Challenging times: Ethics, nursing and the COVID-19 pandemic’, *International Nursing Review* 67(2), 164–167. 10.1111/inr.12598 32578249 PMC7361611

[CIT0018] Valdez, A., 2021, ‘A call to action for 2021’, *Teaching and Learning in Nursing* 16(1), 1–2. 10.1016/j.teln.2020.10.001 33078058 PMC7557298

